# Development of a novel rapamycin loaded nano- into micro-formulation for treatment of lung inflammation

**DOI:** 10.1007/s13346-021-01102-5

**Published:** 2022-02-19

**Authors:** Emanuela Fabiola Craparo, Salvatore Emanuele Drago, Fabiana Quaglia, Francesca Ungaro, Gennara Cavallaro

**Affiliations:** 1 Lab of Biocompatible Polymers, Department of Biological, Chemical and Pharmaceutical Sciences and Technologies (STEBICEF), University of Palermo, Via Archirafi 32, 90123 Palermo, Italy; 2 Lab of Drug Delivery, Department of Pharmacy, University of Napoli Federico II, Via D Montesano 49, 80131 Naples, Italy

**Keywords:** Pulmonary administration, Nanoparticles, Microparticles, Rapamycin, Inflammation

## Abstract

It has recently emerged that drugs such as the mTOR inhibitor rapamycin (Rapa) may play a key role in the treatment of airway inflammation associated with lung diseases, such as chronic obstructive pulmonary disease, asthma, and cystic fibrosis. Nevertheless, Rapa clinical application is still prevented by its unfavorable chemical-physical properties, limited oral bioavailability, and adverse effects related to non-specific biodistribution. In this paper, the design and production of a novel formulation of Rapa based on nano into micro (NiM) particles are detailed. To achieve it, Rapa-loaded nanoparticles were produced by nanoprecipitation of an amphiphilic pegylated poly-ɛ-caprolactone/polyhydroxyethyl aspartamide graft copolymer. The obtained nanoparticles that showed a drug loading of 14.4 wt% (corresponding to an encapsulation efficiency of 82 wt%) did not interact with mucins and were able to release and protect Rapa from degradation in simulated lung and cell fluids. To allow their local administration to the lungs as a dry powder, particle engineering at micro-sized level was done by embedding nanoparticles into mannitol-based microparticles by spray drying. Obtained NiM particles had a mean diameter of about 2-µ, spherical shape and had good potential to be delivered to the lungs by a breath-activated dry powder inhalers. Rheological and turbidity experiments showed that these NiM particles can dissolve in lung simulated fluid and deliver the Rapa-loaded pegylated nanoparticles, which can diffuse through the mucus layer.

**Graphical abstract:**

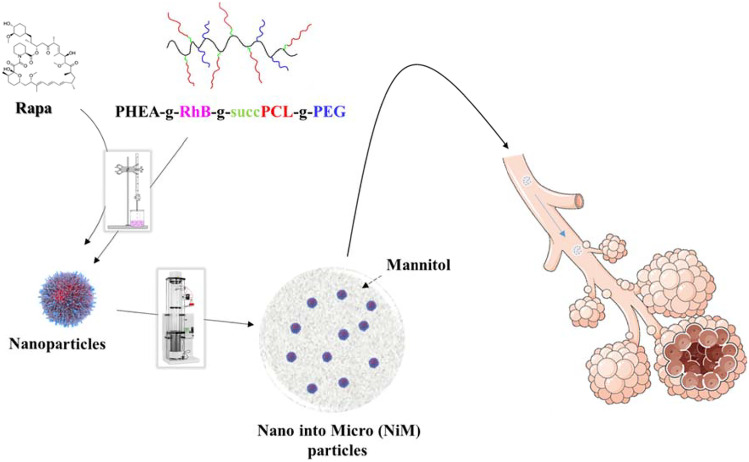

## Introduction

Pulmonary diseases represent a major social and economic problem as many of these are chronic and require multi-drug therapies [1, 2]. Therefore, local pulmonary administration of drugs using innovative formulations with improved bioavailability and easy-to-use devices represents the main objective of many researchers in the field [3–5]. Moreover, the development of advanced inhalable formulations has made it possible to harness new drugs whose administration by other routes is accompanied by poor bioavailability and/or serious side effects due to non-specific biodistribution throughout the body [6, 7]. Meanwhile, innovative formulations may provide a new therapeutic opportunity for lung intervention to established drugs currently used in conventional dosage forms for the treatment of other pathologies [8–10].

Rapamycin (Rapa) is a macrolide whose major cellular target, mammalian target of rapamycin (mTOR), is one of the central regulators of growth, differentiation, metabolism, and survival in many cell types [11]. It is currently approved as an oral solution and/or film-coated tablets for the prophylaxis of organ rejection after transplant, as well as for the treatment of sporadic lymphangioleiomyomatosis (LAM). Moreover, it has been recently repurposed in the treatment of airway inflammation associated with pulmonary diseases such as chronic obstructive pulmonary disease (COPD), asthma, pulmonary arterial hypertension (PAH), and idiopathic pulmonary fibrosis (IPF) [12–14]. Very recently, Rapa is also under evaluation as a drug candidate for optimizing the treatment of coronavirus-induced disease (COVID-19) [15, 16]. Nevertheless, despite the great potential for its use in humans, Rapa applicability is severely limited by formulation problems and poor bioavailability [11].

In literature, there are already numerous attempts to formulate Rapa through innovative formulations such as nanostructured carriers with encouraging results both for the diagnosis and for the treatment of numerous pathologies [17]. Recently, some attempts to realize inhaled formulations of Rapa were reported, with interesting preliminary results [18, 19]. These first results support the concept of local Rapa administration by inhalation to improve drug efficacy in severe lung diseases while minimizing the systemic side effects observed with oral formulations.

Once given the obvious advantage of inhalation to maximize drug bioavailability, formulation/device engineering to ensure correct deposition, drug solubilization in the lung lining fluid, and subsequent absorption is challenging. Particular attention must be given to the materials used for the realization of the aforementioned carriers, by choosing biodegradable and/or biocompatible excipients with tailored properties to control delivery features [20, 21]. Mucus-penetrating nanoparticles can be achieved by decorating the carrier surfaces with suitable materials, such as polyethylene glycols (PEG), able to confer stealth properties and the ability to spread through the mucus present in the airways [22, 23]. Meanwhile, particle engineering at micro-sized level is imperative to meet the requirements allowing their local administration to the lungs as a dry powder by suitable devices, such as with breath-actuated dry powder inhalers (DPIs) [24, 25].

In this work, we develop a dry powder for Rapa inhalation through a nano-into-micro-(NiM) approach [26]. The NiM particles were obtained by spray drying and were constituted of a mannitol (Man) matrix incorporating Rapa-loaded pegylated polymeric nanoparticles. The latter was produced by the nanoprecipitation method starting from a pegylated derivative of the α,β-poly (*N*-2-hydroxyethyl)-dl-aspartamide (PHEA) grafted with a carboxyl-terminated PCL [27]. The properties of the developed NiM particles, in terms of aerodynamic behavior after aerosolization through a DPI and dissolution profile into a physiological medium, were evaluated. Moreover, the capability of the Rapa-loaded nanoparticles, once released due to NiM dissolution, to diffuse through a mucus layer, to protect the entrapped drug from hydrolysis into simulated lung fluid and cell medium, was also investigated.

### Materials and methods

#### Materials

Anhydrous *N*,*N*′-dimethylformamide (a-DMF), anhydrous dimethylacetamide (a-DMA), methanol, diethylether, dichloromethane, poly-ɛ−caprolactone (PCL, ${\overline{M} }_{w}$ = 10–18 kDa), succinic anhydride (SA), dimethylaminopyridine (DMAP), 1,1′-carbonyldiimidazole (CDI), *N*,*N*′-disuccinimidyl carbonate (DSC), diethylamine (DEA), triethylamine (TEA), mannitol, mucin from porcine stomach (type III, bound sialic acid 0.5–1.5%), poly(ethylene oxide) standards, O-(2-aminoethyl)-O’-methyl poly(ethylene glycol) 2000 (H_2_N-PEG) (≤ 0.4 mmol NH_2_/g, 2 kDa), Dulbecco’s phosphate buffer saline (DPBS), fetal bovine serum (FBS), and mannitol (Man) were of analytic grade and obtained from Sigma-Aldrich (Italy). Rapamycin (Rapa) was purchased from Accel Pharmatech (US).

^1^H-NMR spectra were registered by a Bruker Avance II-300 spectrometer, working at 300 MHz (Bruker, Milan, Italy).

Size exclusion chromatography (SEC) analysis was performed by a system from Waters (Mildford, MA) equipped with two columns (Phenogel, 5-μm particle size, pore size: 10^3^ Å and 10^4^ Å) from Phenomenex, and a refractometer. Elution parameters: 50 °C, flow of 0.8 mL/min; eluent: DMF solution of 0.01 M LiBr. Standards: PEGs (range 145–1.5 kDa). Sample preparation: dispersion in the eluent (2.5 mg/mL) and filtration (0.2 μm). Each analysis was conducted in triplicate.

α,β-Poly(*N*-2-hydroxyethyl)-d,l-aspartamide (PHEA) and PHEA-g-RhB were properly synthesized by following procedures already reported in literature [28].

PHEA-g-RhB ^1^H‐NMR (300 MHz, D_2_O, 25 °C, TMS): *δ* 1.15 (12H_RhB_ **CH**_**3**_CH_2_–); *δ* 2.71 (2H_PHEA_ –COCH**CH**_**2**_CONH–); *δ* 3.29 (2H_PHEA_ –NH**CH**_**2**_CH_2_O–); *δ* 3.58 (2H_PHEA_ –NHCH_2_**CH**_**2**_O–); *δ* 4.65 (1H_PHEA_ –NH**CH**(CO)CH_2_–); *δ* 8.00–8.50 (10H_RhB_ **H**-Ar). The ${\overline{M} }_{w}$ of PHEA-g-RhB used in this study was 52.5 Da (${\overline{M} }_{w}/{\overline{M} }_{n}$ = 1.6). The degree of derivatization in RhB (DD_RhB_), determined from the ^1^H-NMR spectra, as reported elsewhere, was equal to 0.6 ± 0.05 mol% [29].

Poly-ɛ-caprolactone-succinate (SUCC-PCL) was synthesized and characterized as reported elsewhere [27, 30].

#### Synthesis and characterization of PHEA-g-RhB-g-SUCC-PCL graft copolymer

A modified synthetic procedure was followed to synthesize PHEA-g-RhB-g-SUCC-PCL graft copolymer with proper derivatization degree (DD%) [30]. Briefly, to an organic PHEA-g-RhB dispersion in a-DMF (33 mg/mL), DEA was added as catalyst according to *R*_2_ = 0.3 that is the molar ratio between DEA and those of repeating units (RUs) of PHEA-g-RhB carrying hydroxyl groups. At the same time, on the basis of *R*_1_ = 3 (that is the mole ratio between CDI and PCL-SUCC), the calculated amount of CDI was added to the organic PCL-SUCC dispersion (66.5 mg/mL in a-DMF), and the resulting mixture was putted at 40 °C for 5 h. After this time, the PHEA-RhB dispersion was added dropwise to that of CDI-activated PCL-SUCC according to the molar ratio between PCL-SUCC and those of PHEA-g-RhB RUs equal to *R*_3_ = 0.12. The resulting mixture was left at 40 °C for 68 h, then the product was recovered by precipitation in diethyl ether, separated from the supernatant by centrifugation (at 4 °C for 15 min, at 9800 rpm), and washed three times with a diethylether:dichloromethane mixture (4:1 v/v). The obtained product was dissolved in DMA, purified by dialysis against water (MWCO 12–14 kDa), and freeze-dried.

^1^H-NMR (300 MHz, [D7].DMF, 25 °C, TMS): *δ* 1.13 (*m*, 12H_RhB_ **CH**_**3**_CH_2_–); *δ* 1.5 and 2.1 (*m*, 6H_PCL_ –[O(O)CCH_2_(**CH**_**2**_)_3_CH_2_]_122_–);δ 2.5 (2d, 2H_PCL_ –[O(O)CCH_2_(CH_2_)_3_**CH**_**2**_]_122_–); δ 2.8 (*m*, 2H_PHEA_ –C(O)CH**CH**_**2**_C(O)NH–); δ 3.2 (*t*, 2H_PHEA_ –NH**CH**_**2**_CH_2_O–); δ 3.50 (*t*, 2H_PHEA_ –NHCH_2_**CH**_**2**_O–); δ 4.3 (*t*, 2H_PCL_ –[O(O)C**CH**_**2**_(CH_2_)_3_CH_2_]_122_–), and δ 5.0 (*m*, 1H_PHEA_ –NH**CH**(CO)CH_2_–); *δ* 7.00–8.00 (*m*, 10H_RhB_ **H**-Ar).

#### PEGylation of PHEA-g-RhB-g-SUCC-PCL graft copolymer

PEGylation of PHEA-g-RhB-g-SUCC-PCL to obtain PHEA-g-RhB-g-SUCC-PCL-g-PEG graft copolymer was done as already reported for similar copolymers [27, 31]. Briefly, to an organic a-DMA dispersion of PHEA-g-RhB-g-SUCC-PCL (64 mg/mL), TEA as catalyst, and DSC were added according to *R*_4_ = 0.1 (the molar ratio between DSC and moles of PHEA RUs carrying hydroxyl groups), and *R*_5_ = 1 (the molar ratio between TEA and moles of DSC). The obtained dispersion was placed to reach at 40 °C. After 4 h, the latter was added dropwise to an organic a-DMA dispersion of H_2_N-PEG (12 mg/mL), according to *R*_6_ = 0.075, being *R*_6_ the molar ratio between H_2_N-PEG and moles of PHEA-g-RhB-g-SUCC-PCL RUs carrying hydroxyl groups. After 18 h at 25 °C, the reaction mixture was purified by dialysis (MWCO 12–14 kDa) against distilled water and freeze-dried to recover the obtained copolymer. PHEA-g-RhB-g-SUCC-PCL-g-PEG graft copolymer was obtained with a yield of 240 wt% based on the starting PHEA-g-RhB-g-SUCC-PCL.

^1^H-NMR (300 MHz, [D7].DMF, 25 °C, TMS): *δ* 1.13 (*m*, 12H_RhB_ **CH**_**3**_CH_2_–); *δ* 1.5 and 2.1 (*m*, 6H_PCL_ –[O(O)CCH_2_(**CH**_**2**_)_3_CH_2_]_122_–); δ 2.5 (2d, 2H_PCL_ –[O(O)CCH_2_(CH_2_)_3_**CH**_**2**_]_122_–); δ 2.8 (*m*, 2H_PHEA_ –C(O)CH**CH**_**2**_C(O)NH–); δ 3.2 (*t*, 2H_PHEA_ –NH**CH**_**2**_CH_2_O–); δ 3.50 (*t*, 2H_PHEA_ –NHCH_2_**CH**_**2**_O–); δ 3.7 (*t*, 4H_PEG_ –[CH_2_**CH**_**2**_O]_44_–); δ 4.3 (*t*, 2H_PCL_ –[O(O)C**CH**_**2**_(CH_2_)_3_CH_2_]_122_–); and δ 5.0 (*m*, 1H_PHEA_ –NH**CH**(CO)CH_2_–); *δ* 7.00–8.00 (*m*, 10H_RhB_ **H**-Ar).

#### Nanoparticle production

Empty or Rapa-loaded pegylated nanoparticles (empty nano-PEG and Rapa-loaded nano-PEG, respectively) were obtained by nano-precipitation. In particular, a PHEA-g-RhB-g-SUCC-PCL-g-PEG graft copolymer dispersion of (1.5% w/v) in DMA (containing or not the drug at a concentration of 0.32% w/v) was placed in a burette and added dropwise to twice-distilled water (1:10 v/v). The mixture was left under stirring for 2 h, dialyzed against twice-distilled water, centrifuged, and filtered, and the obtained nanoparticle dispersion was stored at 5 °C before being used or for further characterization. To obtain empty and drug-loaded non-pegylated nanoparticles (empty and Rapa-loaded Nano), the procedure of nanoprecipitation was followed by using PHEA-g-RhB-g-SUCC-PCL graft copolymer. To obtain PCL-based nanoparticles (PCL nano), the same procedure was followed by using PCL as starting polymeric material.

#### Nanoparticle characterization

##### Size and ζ potential measurements

Hydrodynamic diameter (*Z*-average), polydispersity index (PDI), and ***ζ*** potential of each sample were determined by using a Malvern Zetasizer Nano ZSP instrument (Malvern Instrument, Malvern, UK), a He–Ne laser at λ = 632.8 nm, and at a fixed scattering angle of 175°. Each sample, freshly prepared or dispersed in ultrapure water, was analyzed at 25 °C [30]. Each measurement was repeated in triplicate.

##### XPS analysis

A PHI 5000 VersaProbe II (ULVAC-PHI, Inc.) was used to record XPS spectra of each sample, by using a monochromatic Al-Kα radiation (*hν* = 1486.6 eV) from an X-ray source operating at a spot size of 200 μm, a power of 50 W and an acceleration voltage of 15 kV.

##### Determination of drug loading

The drug loading (DL%), that is the Rapa amount loaded into each drug-loaded sample (nano-PEG or nano), and entrapment efficiency (EE%), expressed as the weight percent ratio between the amount of Rapa actually entrapped into the particles and the theoretical one, were assessed by HPLC analyses. In detail, a Waters Breeze System Liquid Chromatograph system was used, which was equipped with a Luna^®^ C18 column (250 × 4.6 mm, 5 μm, from Phenomenex), an autosampler (40 μL as injected volume), and an UV − vis HPLC detector. Other parameters are as follows: a methanol:water 80:20 v/v solution as mobile phase, a flow rate of 1 mL/min, temperature equal to 25 °C, detection wavelength equal to 277 nm. By plotting peak areas (at retention time = 14 min) versus Rapa standard concentration values in methanol (range of 0.02 − 0.001 mg/mL), a calibration curve was built (*y* = 136,513*x*, *R*^2^ = 0.9994). Each sample was treated as follows: a proper amount was dissolved in DMA, added with methanol (1:9 v/v, 2.5 mg/mL) and the obtained dispersion filtered (with filters at a 0.45-μm pore size). The resulting solution was analyzed by HPLC. Each obtained peak area at 14 min was compared with the calibration curve.

#### Cell viability assay

Cell viability was assessed by a 3-(4,5-dimethylthiazol-2-yl)-5-(3-carboxymethoxyphenyl)-2-(4-sulphophenyl)-2H-tetrazolium (MTS) assay on 16HBE cells, using a commercially available kit (Cell Titer 96 Aqueous One Solution Cell Proliferation assay, Promega) containing MTS and phenazine ethosulfate. 16HBE cells were plated on a 96-well plate at a cell density of 15,000 cells/well in DMEM containing 10% FBS. After 24 h of incubation, the medium was removed, and then, the cells were incubated with 200 μL per well with an aqueous dispersion (DMEM containing 10% FBS) of empty nano-PEG or Rapa-loaded nano-PEG (at concentrations ranging between 0.05 and 0.75 mg/mL). Cell viability in the presence of free Rapa, at concentrations corresponding to those loaded into the Rapa-loaded Nano-PEG sample, was also evaluated. All dispersions were sterilized by filtration using 220-nm filter. After 24- and 48-h incubation, supernatant was removed and each plate was washed with sterile DPBS; after this, cells in each well were incubated with 100 μL of fresh DMEM and 20 μL of a MTS solution, and plates were incubated for 2 h at 37 °C. The absorbance at 490 nm was read using a microplate reader (Multiskan Ex, Thermo Labsystems, Finland). Relative cell viability (percentage) was expressed as (Abs490 treated cells/Abs490 control cells) × 100, based on three experiments. Cells incubated with the medium were used as negative control.

#### Production of NiM particles

NiM formulations were obtained by following a previously reported procedure [26]. In detail, the spray drying process was carried out by using a Buchi Nano Spray Dryer B-90. Liquid feed aqueous dispersions containing Rapa-loaded nano-PEG or Rapa-loaded nano (0.75 mg/mL) and Man (1 g/100 mL) (nanoparticles/Man 1:13 weight ratio) were used to obtain, respectively, NiM_(Rapa/PEG)_ or NiM_(Rapa)_ samples. Before use, each dispersion was sorted through a 1.2-μm filter and then spray-dried with a large spray nebulizer at the inlet temperature of 100 °C. Filtered and dehumidified air was used as the drying gas; the drying gas flow rate was 120 L/min resulting in an inside pressure of 27 mbar with a spray rate of 78% and pump 66%. Each collected NiM sample was appropriately stored at − 20 °C before analysis.

The amount of Rapa loaded into each NiM sample was determined by HPLC. In detail, a known amount of NiM was dissolved in a mixture of DMA and methanol (2.5 mg/mL) for almost 2 h, the obtained dispersion was filtered (0.45 μm), and the supernatant was analyzed by HPLC following the method reported above.

#### NiM characterization

##### SEM and OM analyses

NiM_(Rapa/PEG)_ sample was laid on a double-sided adhesive tape, previously applied on a stainless steel stub, which was then sputter-coated with gold prior to microscopy examination, and then observed by using by using a Phenom™ ProX Desktop SEM microscope.

The OM analysis was conducted by recording transmittance images of NiM_(Rapa/PEG)_ dispersed in paraffin oil with a ZEISS optical microscope, using the AxioVision software.

The ImageJ program was used to calculate the average diameter of each sample from either SEM or OM images by analyzing a sufficiently representative number to constitute a certain datum (> 500 particles).

##### Drug stability

The Rapa stability was evaluated in physiologic conditions mimicking fluid by quantifying the amount of intact drug over time. In particular, a known amount of Rapa (0.09 mg), free or loaded into NiM_(Rapa/PEG)_, was dispersed in 30 mL of simulated lung fluid (SLF4) (MgCl_2_ (0,2033 g/L), NaCl (6.0193 g/L), KCl (0.2982 g/L), Na_2_SO_4_ (0.0710), CaCl_2_ dihydrate (0,3676 g/L), sodium acetate (0.9526 g/L), NaHCO_3_ (2.6043), sodium citrate dihydrate (0.0970 g/L), NaH_2_PO_4_ monohydrate (0.1420), dipalmitoylphosphatidylcholine (DPPC, 0,02 w/v%)), or cell medium (Dulbecco’s phosphate-buffered saline (DPBS):fetal bovine serum (FBS) (90:10 v/v) mixture) [32]. At fixed times (0, 1, 2, 4, 7, 12, 16, and 24 h), each dispersion was freeze-dried and properly treated in order to recover the intact drug. For free Rapa stability, each freeze-dried sample was treated with 7 mL of methanol, while for Rapa-loaded NiM_(Rapa/PEG)_, the freeze-dried sample was treated with 2 mL of DMA and 5 mL of methanol. Then, the obtained organic dispersions were centrifuged and the supernatants were analyzed by HPLC.

##### Drug release

The Rapa release profile was evaluated in physiologic conditions mimicking fluid by quantifying the amount of intact drug released from the sample over time. In particular, a known amount of Rapa (0.09 mg) loaded into NiM_(Rapa/PEG)_, was dispersed in 30 mL of simulated lung fluid (SLF4), or cell medium [32]. At fixed times (0, 1, 2, 4, 7, 12, 16, and 24 h), each dispersion was ultra-centrifuged; the supernatant was freeze-dried and properly treated in order to recover the intact drug. In particular, the freeze-dried sample was treated with 1.5 mL of methanol. Then, the obtained organic dispersions were centrifuged, and the supernatants were analyzed by HPLC.

#### NIM aerodynamic behavior

The aerosolization properties of NiM_(Rapa/PEG)_ were tested after delivery from breath-activated reusable DPIs working with single unit capsule containing the dry powder using a next-generation impactor (NGI) (Copley Scientific, UK) according to Ph. Eur. 10th Ed. Two devices with different resistances to the airflow were tested: the low-resistance DPI RS01 (Plastiape, Italy) and the medium-resistance DPI TurboSpin^®^ (PH&T Pharma, Italy. For each test, a hard gelatin capsule (size 2, Capsugel, USA) was filled with about 20 mg of the powder and placed in the DPI. The NGI was activated at 60 or 90 L/min, respectively, for TurboSpin^®^ and RS01.

The powder deposited on the seven NGI collection cups, in the induction port and in the micro-orifice collector (MOC), was quantitatively recovered by dissolution in an appropriate amount of DMF. The amount of NiM in the samples was determined by spectrofluorimetric analysis at λ_ex_ = 520 nm. A calibration curve was derived from serial dilutions of a standard solution of fluorescent NiM (2.5 mg/mL) in DMF (0.0125–1.25 mg/mL concentration range, *R*^2^ ≥ 0.99).

The experimental mass median aerodynamic diameter (MMAD_exp_) was calculated according to Ph.Eur. deriving a plot of cumulative mass of powder deposited in each collection cup versus cut-off diameter of the respective stage. The fine particle fraction (FPF) was calculated considering the actual amount of NiMs deposited on stages with MMAD < 5 μm as compared to the initial amount loaded into the DPI (nominal dose of NiMs). The respirable fraction (RF) was defined as the percentage of NiMs deposited on stages with MMAD < 5 μm as compared to the total dose of NiMs deposited in the NGI.

#### Interaction with artificial mucus

##### Rheological analysis

Measurements of interactions between each chosen sample and mucin were determined by rheological analysis at the temperature of 37 °C by using a rheometer (TA Instruments) equipped with concentric cylinders geometry. A strain sweep (5–30%) was performed on mucin dispersion at 1.0 Hz to determine the linear viscoelastic region, which was found to be in the range of 10–20%. Then, a time sweep (30 min) was performed for all samples at 15% constant strain and 1.0 Hz constant frequency to determine complex viscosity (η*). Chosen samples were as follows: NiM_(Rapa/PEG)_ (corresponding to 14 mg of Rapa-loaded nano-PEG), NiM_(Rapa)_ (corresponding to 14 mg of Rapa-loaded nano), free Rapa (at a concentration corresponding to the drug loaded into the nanoparticles), and chitosan as positive control. For the analyses of mucin-sample mixture, a certain amount of each sample was added to 14 mL of mucin dispersion in PBS (1 mg/mL) and mixed gently with a spatula for 20 s. Obtained dispersion was loaded in the rheometer and then equilibrated to 37 °C for 20 min. To prevent dehydration during rheological measurements, a solvent trap was placed on the top of the geometry.

##### Turbidimetric analysis

Measurements of interactions between nanoparticles and mucin were determined by turbidimetry. A proper amount of NiM, corresponding to 0.2 mg of Nano-PEG were dispersed in 190 µL of PBS, were mixed with 10 µL of mucin dispersion at the concentration of 20 mg/mL in PBS. Analyzed samples were as follows: NiM_(Rapa/PEG)_, NiM_(Rapa)_, and free Rapa (at the concentration corresponding to the drug loaded into the nanoparticles). After incubation at 37 °C, the turbidity was measured each 50 min until 6 h. The transmittance at the *λ* of 570 nm was recorded by microplate reader (Multiskan Ex, Thermo Labsystems, Finland). The value obtained from a mucin-free dispersion of each sample was subtracted from each transmittance value. Data were expressed as a percentage ratio between the transmittance of the sample and the transmittance of the 1 mg/mL mucin dispersion.

##### Muco-diffusion assay

The capability of the Nano-PEG to diffuse through a mucin dispersion was evaluated using a diffusion test, as reported elsewhere [26]. Sixty milligrams of agarose was dispersed in 20 mL of distilled water, and the dispersion was heated until a clear solution was obtained; 1.3 mL of this dispersion was deposited in several 4-mL vials, allowed to harden at room temperature and stored at 4 °C until use. Subsequently, 2 mL of mucin dispersion (1 mg/mL in PBS) was placed on the hardened agarose gel, and 600 μL of a NiM_(Rapa/PEG)_ or NiM_(Rapa)_ aqueous dispersion (5 mg/mL) was placed on the mucin layer and incubated at 37 °C. At regular time intervals (2, 4, 6, 8, and 24 h), the mucin layer containing each sample was removed; the remaining agarose gels were rinsed three times with 2 mL of distilled water, dissolved at 60 °C, and analyzed by UV spectrophotometry, at the *λ* of 561 nm.

#### Statistical analysis

All the experiments were repeated at least three times. All data are expressed as means ± standard deviation. All data were analyzed by Student’s *t* test. A *p*-value < 0.05 was considered statistically significant, while a *p*-value < 0.01 was considered highly significant.

## Results and discussion

In this paper, a novel inhalable formulation for repositioning of rapamycin (Rapa) was developed for the management of inflammation-based lung diseases such as asthma and chronic obstructive pulmonary disease (COPD). In particular, a powder product was obtained by following the nano-into-micro-(NiM) approach, which comprises the incorporation of Rapa-loaded polymeric nanoparticles into sugar-based microparticles to be administered by dry powder inhalers (DPIs). Upon inhalation, these microparticles can dissolve releasing the nanostructured carriers, which will protect and deliver the drug cargo through the mucus layer until the bronchial epithelium.

## Design and development of polymeric nanostructured carriers for Rapa

**Fig. 1:**
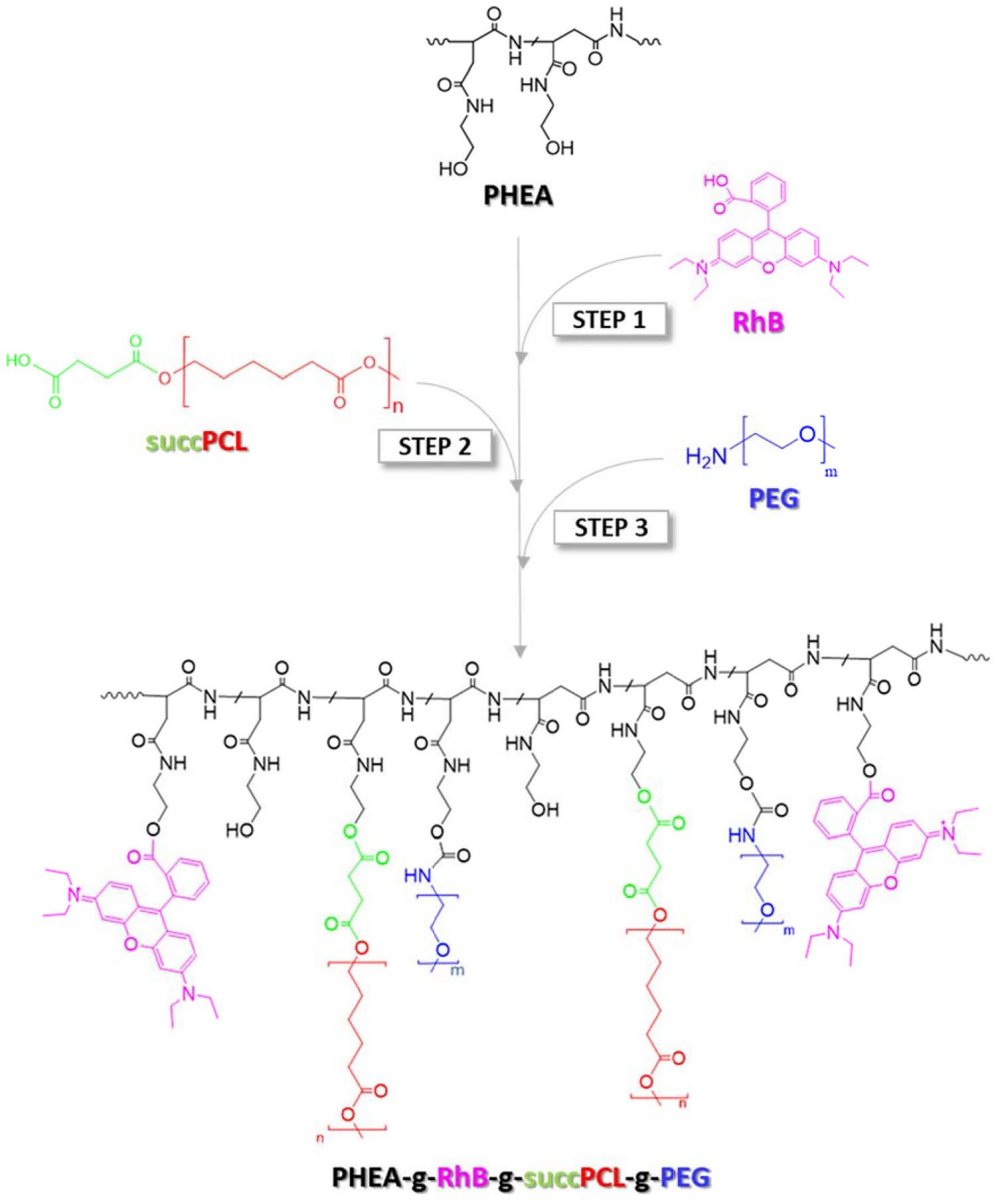
The synthetic route of PHEA-g-RhB-g-SUCC-PCL-g-PEG graft copolymer (*n* = 122, *m* = 44). Reagents and conditions: (step 1) a-DMF, CDI, DEA, 4 h at 40 °C, 48 h at 40 °C; (step 2) a-DMF, CDI, DEA, 5 h at 40 °C, 68 h at 40 °C; (step 3) a-DMA, DSC, TEA, 4 h at 40 °C, 18 h rt

To realize the nanostructured carrier, a polymeric material with proper functional and structural properties suitable for pulmonary administration was designed and synthesized by a modification of a synthetic procedure already described [27]. In particular, on the α,β-poly(*N*-2-hydroxyethyl)-dl-aspartamide (PHEA), rhodamine B (RhB), the succinylated derivative of poly-ɛ-caprolactone (PCL-SUCC), and the polyethylene glycol (PEG), were sequentially grafted, obtaining the PHEA-g-RhB-g-SUCC-PCL-PEG. To design nanoparticles suited for inhalation, each component was properly chosen. PHEA, the main protein-like polymer chain, was chosen due to its biocompatibility, essential for pulmonary administration, and for the fact that offers the opportunity to graft various functionalities due to the presence of a reactive group on each repeating unit [29, 33]. The RhB was chosen as fluorescent dye and covalently linked on the PHEA backbone to make it stably fluorescent without modifying its self-assembly characteristics, making it detectable and quantifiable by spectroscopic techniques [34]. PCL-SUCC was grafted on the PHEA-g-RhB to endow the resulting copolymer with suitable amphiphilic properties and to make it an excellent starting material for the nanoparticle production, able to entrap hydrophobic drugs such as Rapa. The PEG was chosen as a further material to be grafted on the PHEA backbone to increase the hydrophilicity of the nanoparticle surface, thus giving stealth properties and the ability to spread through the mucus present in the airways [22, 23]. Last but not least, all the selected polymeric materials represent valid candidates to be used for the realization of safe carriers for inhalation as their in vitro-in vivo biocompatibility has already been demonstrated [27, 29]. The synthetic steps and chemical structure of the PHEA-g-RhB-g-SUCC-PCL-g-PEG graft copolymer are reported in Fig. 1.

The degree of derivatization in RhB (DD_RhB_), PCL (DD_PCL_), and PEG (DD_PEG_) was determined from the ^1^H-NMR spectra, as reported elsewhere, and result to be equal to 0.6 ± 0.05 mol%, 3.4 ± 0.1 mol%, and 4.3 ± 0.06 mol%, respectively [27, 31]. Characterization by size exclusion chromatography (SEC) confirmed the occurrence of the conjugation reactions, being the ${\overline{M} }_{w}$ equal to 182 kDa (${\overline{M} }_{w}/{\overline{M} }_{n}$ = 1.28).

Then, starting from the PHEA-g-RhB-g-SUCC-PCL-g-PEG graft copolymer, the nanoprecipitation method was followed to obtain polymeric pegylated nanoparticles (empty nano-PEG). Experimental parameters and condition to carry out the nanoprecipitation are reported in detail in the experimental part.

Freshly prepared nanoparticle dispersions underwent size and surface analyses. Both empty and Rapa-loaded nano-PEG were smaller than 100 nm, with a negative ζpotential (Table 1).

**Table 1:** Mean size, polydispersity index (PDI), and *ζ*-potential of freshly prepared empty and Rapa-loaded nano-PEG in bidistilled water

**Sample**	***Z*-average (nm)**	**PDI**	**ζ potential (mV ± SD)**
**Empty nano-PEG**	54.7	0.34	− 16.7 ± 7.8
**Rapa-loaded nano-PEG**	51.1	0.20	− 14.4 ± 4.6

As previously stated, the choice to graft PEG chains on the starting copolymer stems from the idea that the nanoprecipitation could produce nanoparticulate systems with PEG chains exposed on the surface, which could favor the diffusion through the mucus layer. Therefore, the surface chemical composition of PHEA-g-RhB-g-SUCC-PCL-g-PEG-based nanoparticles (named empty nano-PEG sample) was analyzed to evaluate the surface pegylation through the X-ray photoelectron spectroscopy (XPS) technique. To better highlight the presence of PEG on the surface of the nano-PEG particles and the extent of this pegylation, the XPS analysis was also carried out on nanoparticles obtained starting from PCL alone (named empty PCL nanosample), or from PHEA-g-RhB-g-SUCC-PCL copolymer (named empty nano sample).

The XPS analysis of the investigated samples is reported in Table 2 as the relative distribution of the carbon, oxygen, and nitrogen species on the particle surface, determined by a curve-fitting procedure of the photoelectron peaks. It is evident that the C 1 s relative atomic percentage on the empty PCL nano sample is higher than that found on empty nano and empty nano-PEG samples, that are both PHEA derivative-based nanoparticles, where instead it is present nitrogen, at binding energy equal to 399.7 eV. It should be attributed to N–C/N–C(O) linkage present in the PHEA backbone and, as expected, is absent in the PCL-based sample (empty PCL Nano). This result indicates that on the empty nano and empty nano-PEG sample surface is exposed PHEA backbone [27].

**Table 2:** XPS Surface chemical composition of obtained nanoparticles^a^

**Nanoparticle sample**	**C 1 s**	**O 1 s**	**N 1 s**
**Empty PCL nano**^**b**^	74.49	25.51	–- 3.90
**Empty nano**	69.70	26.40	
**Empty nano-PEG**	72.60	25.32	2.08

^a^Relative distribution of the carbon, nitrogen, oxygen, and phosphorus species on the nanoparticle surface determined by a curve-fitting procedure of the photoelectron peaks

^b^Nano obtained from PCL alone by nanoprecipitation

To obtain more detailed information on the type of bond that affects carbon, the relative distributions of the carbon species on the nanoparticle surface were determined by a curve-fitting procedure, and results are reported in Table 3.

**Table 3:** XPS Surface chemical composition of obtained nanoparticles^a^

		**C–C/C–H**	**C–N**	**C–O**	**N–C = O**	**OC = O**
**Nanoparticle sample:**	**BE (eV)**	**284.8**	**286.0**	**286.2**	**287.6**	**288.6**
**Empty PCL nano**^**b**^		66.6	–-	16.6	–-	16.8
**Empty nano**		57.1	6.3	16.6	6.6	13.4
**Empty nano-PEG**		39.1	4.5	42.9	4.3	9.2

^a^Relative distribution of the carbon species on the nano surface determined by a curve-fitting procedure of the photoelectron peaks

^b^Nano obtained from PCL alone by nanoprecipitation

As expected, in both the empty nano and empty nano-PEG samples, the C 1 s core level spectrum showed species at the binding energy (BE) of 286.0 and 287.6 eV, attributed respectively to C–N and N–C = O bonds (belonging to PHEA backbone), that are absent in the empty PCL-based sample. Very interestingly, the C 1 s core level spectrum at the BE of 286.2 eV, is more than double in the empty nano-PEG than in the other samples (42.9 vs. 16.6%); since this level is assigned to C–O–C bonds belonging to PEG chains, it demonstrates the presence of PEG on these nanoparticles [26].

**Fig. 2:**
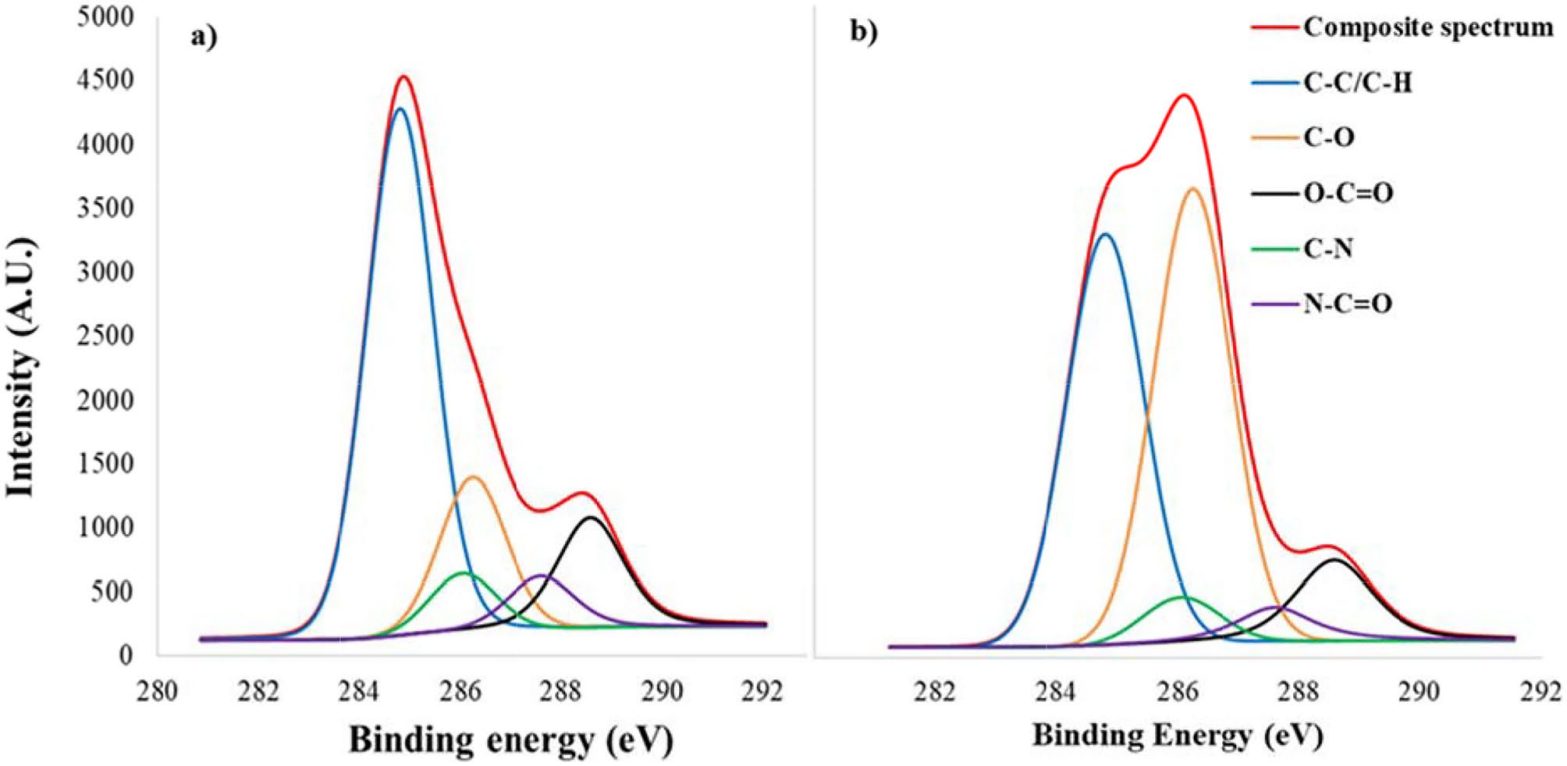
Curve-fitting of the C 1 s spectrum of (**a**) empty nano and (**b**) empty nano-PEG samples

In Fig. 2(a and b), the curve fittings of the C 1 s spectra of empty nano and nano-PEG samples are reported, respectively. As expected, in these graphics, the C 1 s core level spectrum at the BE of 286.2 eV of the nano-PEG was significantly higher and mainly represented than that found on the spectrum of nano.

From the characterization of empty systems, it emerged that nano-PEG particles are of adequate size and expose the PEG on the surface; therefore, Rapa was entrapped into these particles simultaneously with the nanoparticle formation by dissolving the drug in the organic phase in the nanoprecipitation process. In this way, the drug-loaded pegylated nanoparticles (Rapa-loaded nano-PEG sample) were produced, whose mean size and ζ potential values are not affected by the presence of Rapa.

The amount of entrapped drug, reported as both drug loading (DL%) and entrapment efficiency (EE%), was quantified by HPLC analysis and reputed to be, respectively, 14.4 wt%, and 82 wt%.

## Biological assay

**Fig. 3:**
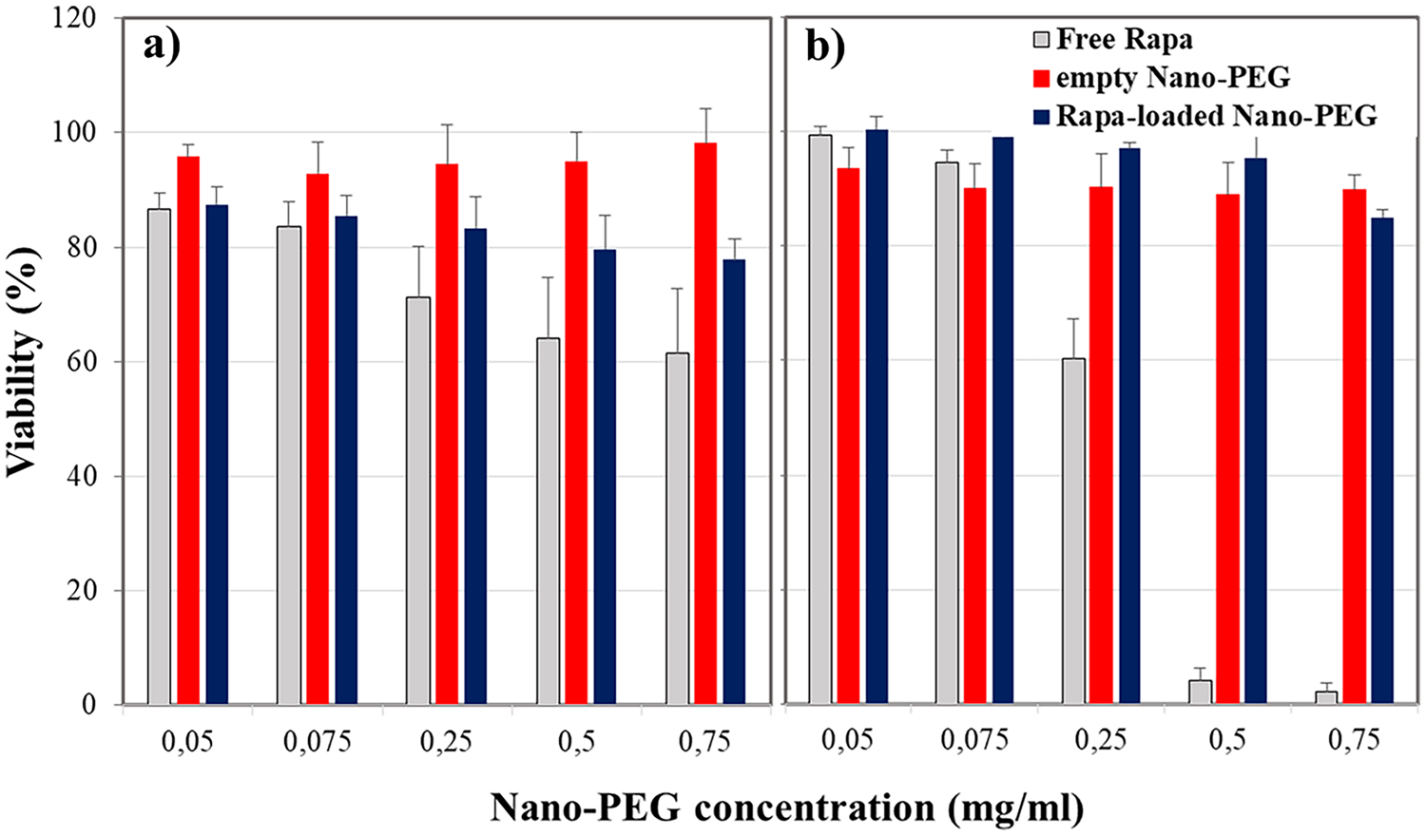
16-HBE viability % after incubation in the presence of empty or Rapa-loaded nano-PEG in the range 0.05–0.075 mg/mL, after 24 h (**a**) and 48 h (**b**)

Conceiving the Rapa-loaded nano-PEG for the local treatment of inflammation associated with COPD and asthma, biocompatibility has been evaluated on human bronchial epithelial cells (16-HBE). As can be seen in Fig. 3, both empty nano-PEG and Rapa-loaded nano-PEG have no significant effects on cell viability after 24- and 48-h incubation, being the cell viability always higher than 80%, at chosen experimental conditions. At the same time, the free drug shows a significant cytotoxicity dependent on either dose or incubation time. This result could be explained considering that, when cells are incubated with Rapa-loaded nanoparticles, the released drug concentration is lower than when incubated with free drug due to the drug release kinetics. Therefore, it can be stated that the lower toxicity could be mainly due to the lower concentration of intact drug present in the incubation medium.

## Development of Rapa-loaded NiM dry powders

**Fig. 4:**
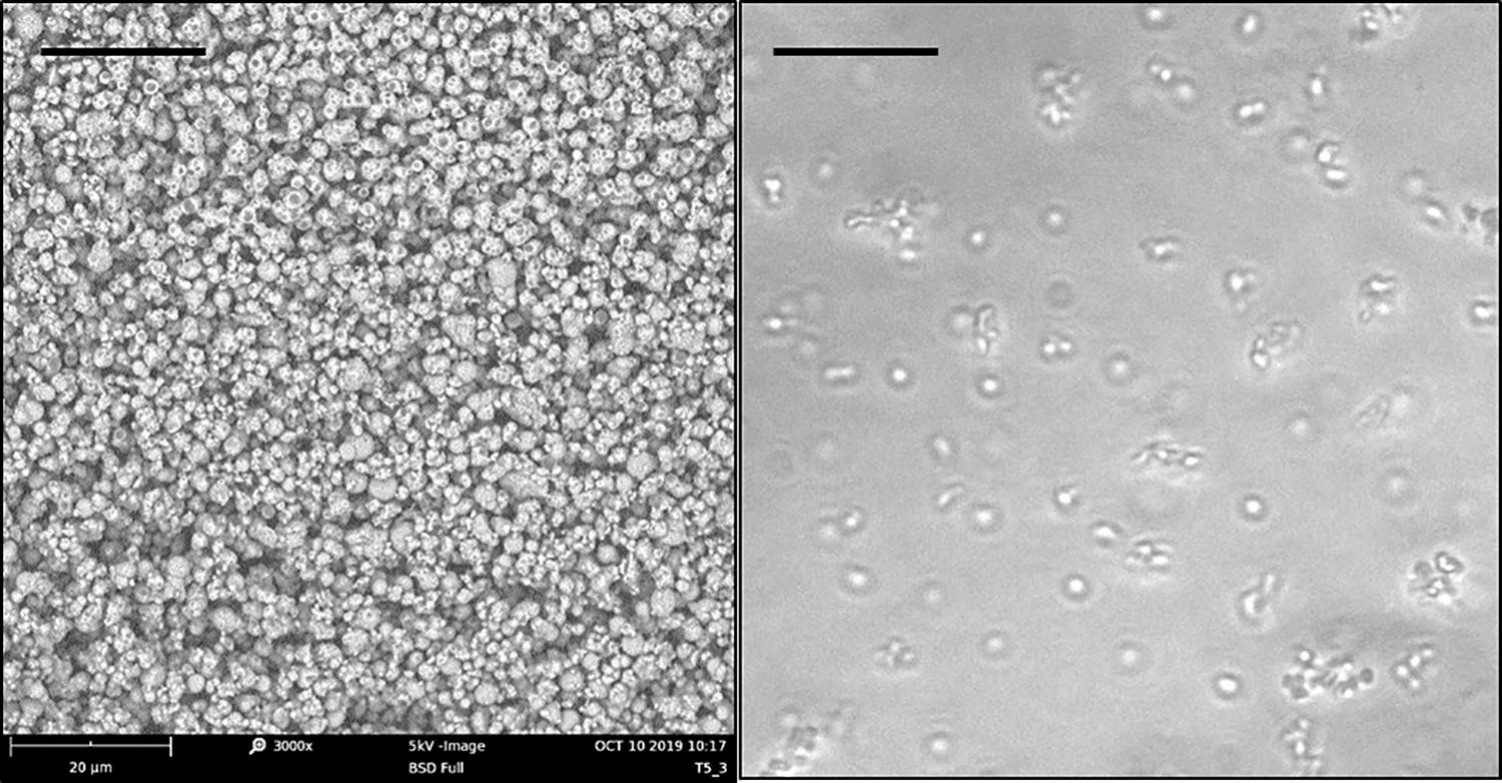
SEM and OM images of NiM_(Rapa/PEG)_ particles. The bar represents 20 μm

To turn nanoparticles into inhalable dry powders, Rapa-loaded nano-PEG particles were entrapped into micrometer particles of mannitol (Man). The latter was chosen because it is a largely used pharmaceutical excipient as well as a therapeutically active and safe substance for inhalation, being able to vary the viscoelastic properties of the mucus and to increase the hydration of the periciliary fluid layer [26]. Microparticles, named NiM_(Rapa/PEG)_, were obtained by spray drying (SD), by following the experimental conditions and parameters described in the experimental part, with a yield of 67 wt%. After production, the morphology and diameter were investigated by combining scanning electron microscopy (SEM) (analyzed as powder), and optical microscopy (OM) analyses. Representative images are reported in Fig. 4.

The mean diameter of obtained NiM_(Rapa/PEG)_ particles was calculated from SEM, and OM images with the help of the software ImageJ were almost comparable and resulted to be, respectively, 1.53 ± 0.26 μm and 1.71 ± 0.36 μm.

NiM_(Rapa/PEG)_ particles were dispersed in water, and the obtained nanoparticle dispersion was characterized in terms of the mean size, which resulted to be tripled compared to freshly prepared. Therefore, the powder formulation, when in contact with an aqueous medium, released empty and Rapa-loaded nano-PEG with mean size lower than 200 nm and ζ potential values comparable with the fresh nanoparticle dispersion. In particular, mean sizes were equal to 191.5 and 162.1 nm, while ζ potential values equal to − 13.8 ± 7.7 and − 12.2 ± 9.6, mV respectively, for empty and drug-loaded nano-PEG. Although a size increase is observed after SD, it is acceptable considering that the mean size is still below 200 nm, therefore potentially able to spread in the mucus [23]. Moreover, the absence of thermal degradation phenomena on both the drug and the copolymer due to the SD process was evaluated by HPLC and SEC analyses, respectively (data not shown).

Having a macrolide structure, Rapa undergoes chemical instability in physiological fluids [35]. Therefore, the entrapment into nano-PEG could protect the drug from degradation phenomena and allow a controlled release of the entrapped drug, maintaining a proper drug concentration in the administration site.

**Fig. 5:**
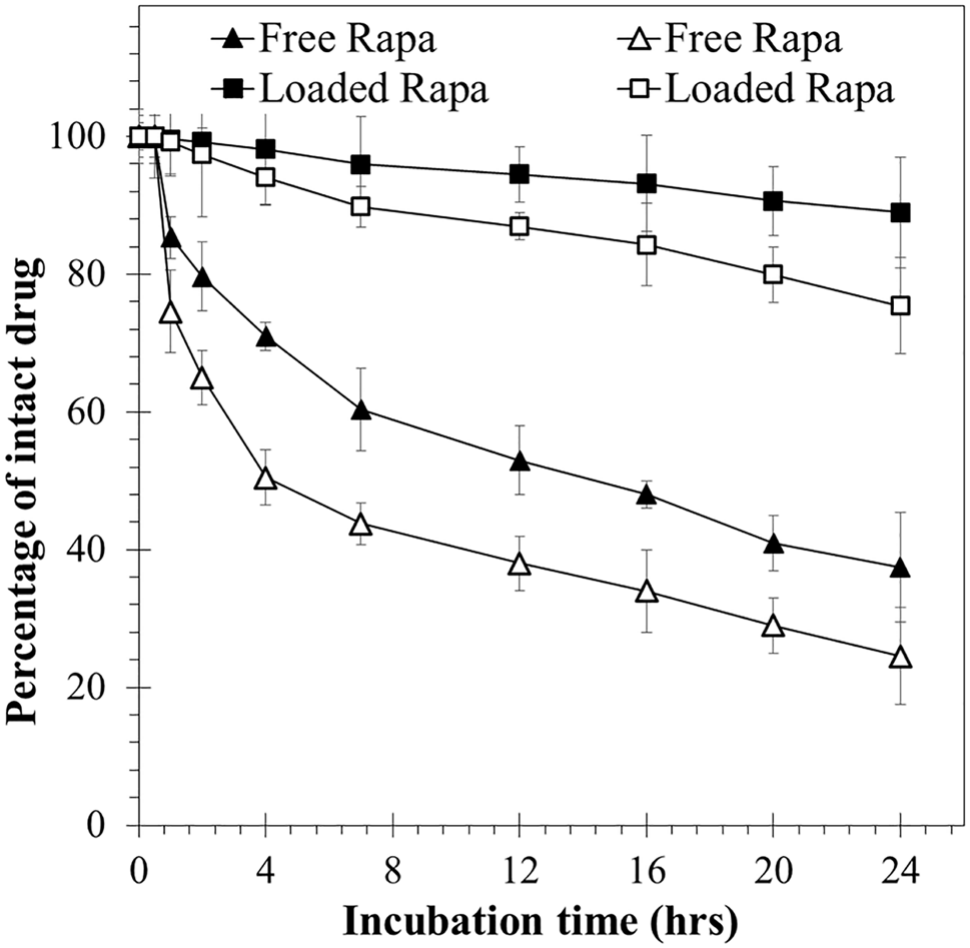
Stability of free Rapa (triangle) or loaded into NiM_(Rapa/PEG)_ (square), in simulated lung fluid (full indicator), and cell medium (empty indicator), at pH 7.4. Data represent mean ± SD (*n* = 3)

To evaluate the effect of Rapa entrapment into nano-PEG on the drug degradation, a stability study was carried out in simulated lung fluid or cell medium (Dulbecco’s phosphate-buffered saline (DPBS): fetal bovine serum (FBS) (90:10 v/v) mixture) [30, 32]. The presence of FBS increases the drug solubility as well as the degradation, compared to saline medium alone [36, 37]. In particular, the free drug or a Rapa-loaded nano-PEG dispersion (obtained by dispersion of a proper NiM_(Rapa/PEG)_ amount) were dispersed in each medium, and, at fixed time intervals, the total amount of intact drug was quantified by HPLC. Results are reported in Fig. 5.

After 24 h, the incubation of free Rapa in simulated lung fluid or cell medium resulted in a reduction of the amount of intact drug down to 40 and 25 wt%, respectively (triangles in Fig. 5). At the same time, the amount of intact drug found in each medium upon incubation of NiM_(Rapa/PEG)_ was equal to 90 and 75 wt% of the total entrapped drug, in simulated lung fluid or cell medium respectively (squares in Fig. 5). It can be reasonably stated that Rapa loading into the nanoparticulate matrices protects it from degradation in both simulated media.

This result supports the hypothesis that the incorporation of Rapa into a carrier can improve its stability profile, which currently causes significant changes in the bioavailability of the marketed oral dosage form. Furthermore, the possibility of administering these nanoparticles by inhalation, thanks to the NiM strategy, could optimize the bioavailability of the drug into the lungs.

**Fig. 6:**
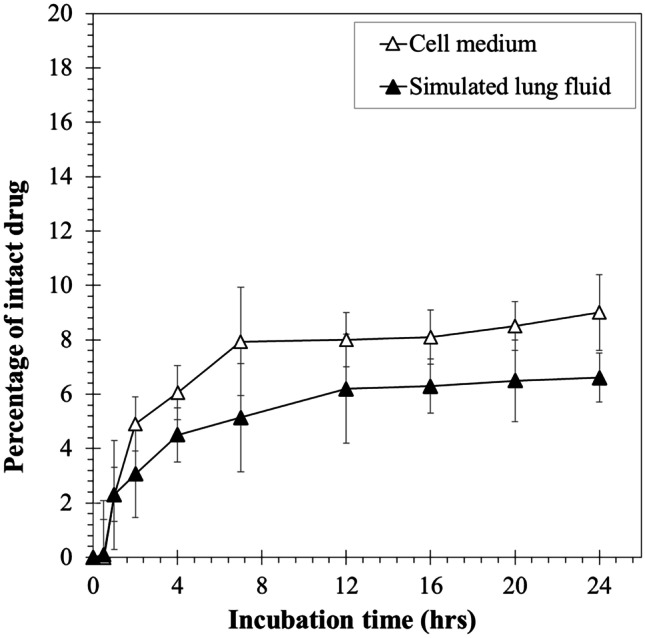
Rapa release profile in simulated lung fluid (full indicator), and cell medium (empty indicator), at pH 7.4. Data represent mean ± SD (*n* = 3)

To evaluate the fraction of intact Rapamycin in the incubation media, on the NiM_(Rapa/PEG)_ sample, a drug release study was also carried out in simulated lung fluid and cell medium. In particular, the intact drug released in each incubation medium was quantified over time. Obtained data are reported in Fig. 6.

Results showed that Rapa is released from the nanoparticles in a controlled way, and that, although the total drug amount in the release medium is lowered by degradation phenomena, a constant amount of intact drug is maintained over time.

## Rapa-loaded NiM aerosol performance

**Fig. 7:**
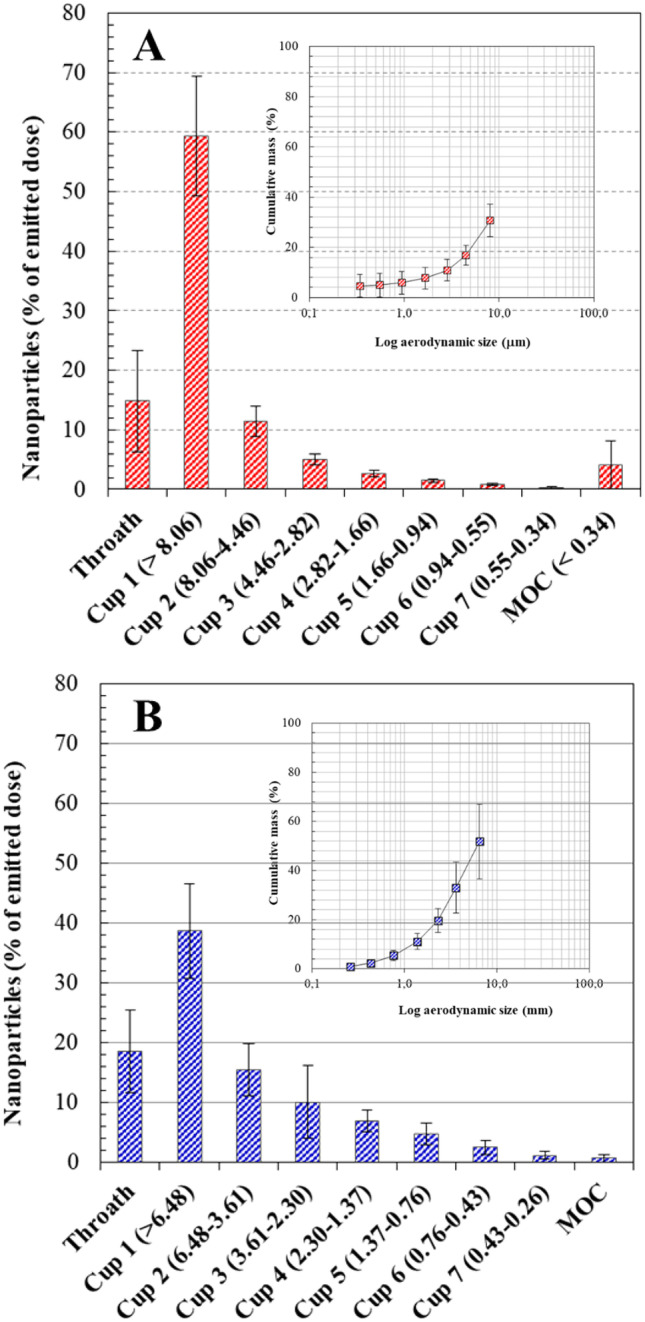
In vitro aerosol performance of NiM_(Rapa/PEG)_ upon delivery by the breath-actuated DPIs: **A** TurboSpin^®^ (60 L/min). **B** RS01 (90 L/min). Main panels: NGI deposition patterns. Insets: cumulative mass recovered as a function of the cutoff diameter of the respective NGI stage

The main driving forces affecting the performance of a DPI are the inspiratory flow generated by the patient, and the turbulence produced inside the device, which depends on its intrinsic technical characteristics. The aerosol performance of NiM_(Rapa/PEG)_ was investigated upon delivery from either a medium-resistance (TurboSpin^®^) or a low-resistance (RS01) DPI. Since in the case of medium-resistance DPIs, a limited dependence of powder aerosolization on the airflow rate is expected, and aerosol performance was evaluated at 60 L/min. On the other hand, the intrinsic low-resistance of RS01 required a higher airflow rate to aerosolize the powder, that is 90 L/min. Results are shown in Fig. 7 as percentage of emitted dose deposited on the NGI cups and cumulative mass recovered as a function of the cutoff diameter. As expected, the NGI deposition pattern of NiM dry powders varied as a function of the DPI resistance. When delivered through TurboSpin^®^ at 60 L/min flow rate (Fig. 7A), more than 70% of the dry powder sample was recovered from throat and cup 1 (MMAD > 8.06 μm), suggesting poor aerosolization properties. In fact, NiM_(Rapa/PEG)_ particles displayed an FPF (i.e., particles with an MMAD lower than 4.46 μm) of 10.6 ± 2.8% and an RF of 16.8 ± 2.5%. Since the cumulative mass aerosolized was limited (ca. 30%), the value of MMAD_exp_ could not be determined.

The deposition pattern of the NiM_(Rapa/PEG)_ dry powder improved when using RS01 (Fig. 7B). In fact, the amount of NiM found in the throat and cup 1 of the NGI lowered down to the benefit of cups 2–7 and MOC (cutoff diameter < 6.48 μm), where more than 45% of the recovered dose was deposited. In turn, an RF as high as 42.5 ± 4.98% was calculated. The MMAD_exp_ was 7.09 ± 1.25 μm, suggesting a good potential for bronchial deposition of the developed NiMs. Overall, results suggest that NiM is better suited to a low resistance DPI.

## NiM interactions with the mucus barrier

From what has been reported so far, the followed strategy made it possible to obtain NiM_(Rapa/PEG)_, consisting in pegylated Rapa-loaded nanoparticles embedded into Man microparticles. The latter, once placed in contact with fluids that mimic biological ones, can dissolve to reconstitute a dispersion of nanoparticles, capable in turn of protecting the drug. However, it must be also considered that, to reach the bronchial epithelium, these particles must spread through a layer of mucus, which can vary in thickness and composition (i.e., in mucin concentration) according to the disease and its severity.

Therefore, to evaluate the possible interactions with the components of the mucus, which would be unfavorable for the diffusion of particles through it, rheological measurements were carried out.

**Fig. 8:**
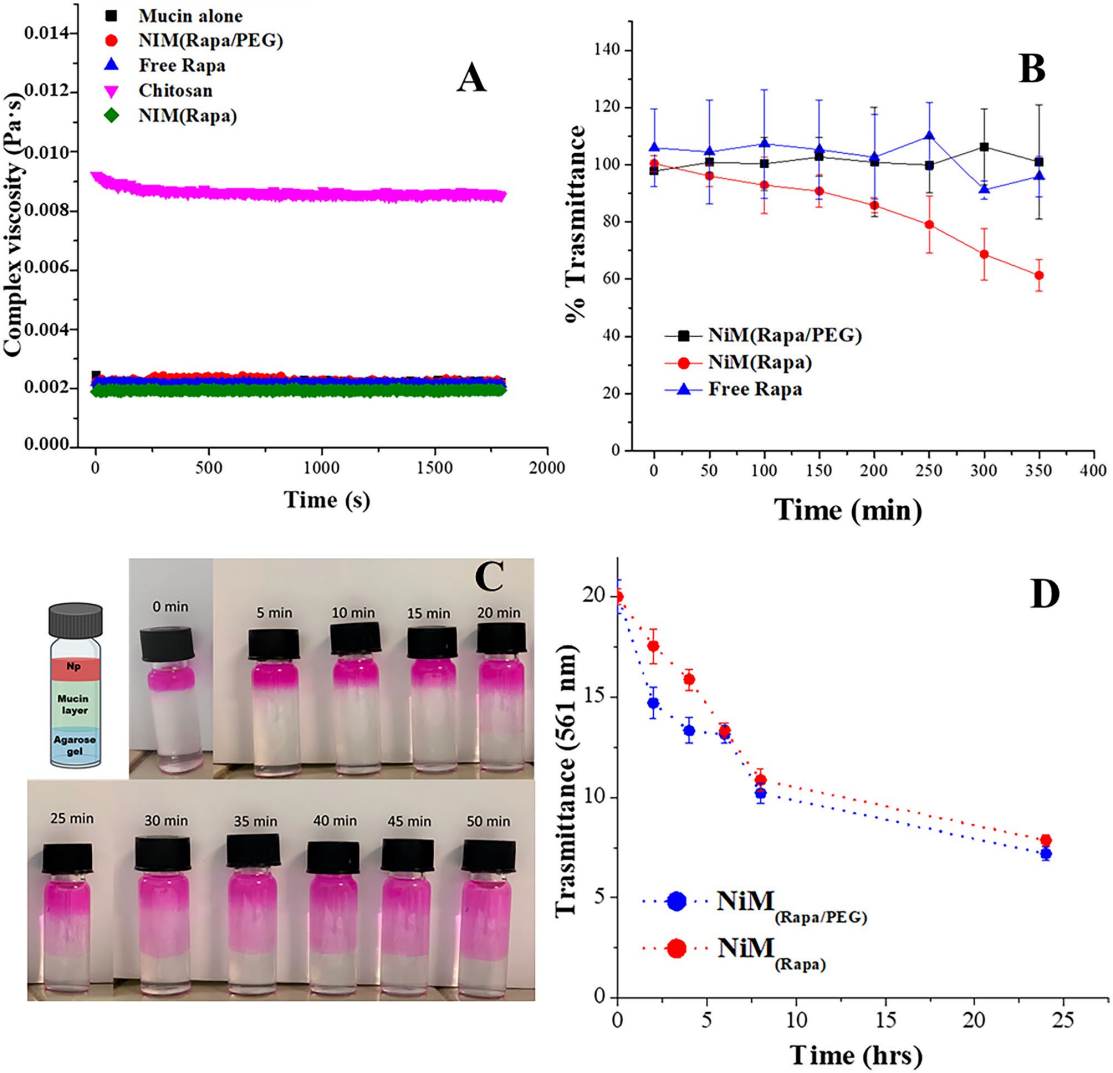
**A** Complex viscosity (η*) of mucin dispersion treated with NiM_(Rapa/PEG)_ and NiM_(Rapa)_ particles, Rapa (at a concentration value corresponding to the drug loaded into the NiM_(Rapa/PEG)_, after 1 h incubation at 37 °C. η* of untreated mucin dispersion and treated with chitosan is used as negative and positive control, respectively. **B** Turbidity values of NiM_(Rapa/PEG)_, NiM_(Rapa)_, free drug (at a concentration lower or higher the drug solubility), incubated with a mucin dispersion (1 mg/mL). **C** Muco-diffusion test of Rapa-loaded nano-PEG through a mucus layer. **D** Transmittance at λ 561 nm of agarose gel recovered after different incubation times with NiM_(Rapa/PEG_) and NiM_(Rapa)_

In particular, NiM_(Rapa/PEG)_ particles were incubated with a mucin dispersion in PBS, and the complex viscosity was measured for 30 min. The experiment was also repeated in the presence of NiM_(Rapa)_ and in the presence of free drug (at a concentration corresponding to that carried inside NiM_(Rapa/PEG)_). Mucin alone and chitosan were used respectively as the negative and positive control. Data are reported in Fig. 8A.

As can be seen, no significant difference was found in the rheological behavior between mucin alone and mucin containing NiM particles (pegylated or not) or free Rapa, while chitosan dispersion in mucin shows a significantly higher complex viscosity compared to all the other samples.

As well known, the interaction capacity of polymers with mucus can also be evaluated through turbidity measurements, since polymer-mucin interactions would result in a reduction of sample transmittance. Therefore, transmittance measurements at λ = 570 nm were carried out on sample-mucin dispersions as a function of incubation time (Fig. 8B).

As can be seen, NiM_(Rapa/PEG)_ particles have a low tendency to interact with mucin independently of the incubation time. On the contrary, NiM_(Rapa)_ interact with mucin as the incubation time increases, as the transmittance reduces over time. Data confirm that the surface pegylation confers to the nanoparticles the potential capability to diffuse through the mucus. Moreover, the absence of interactions of free Rapa with mucin was also demonstrated.

The capability of NiM_(Rapa/PEG)_ to diffuse through the mucus layer was also confirmed by a muco-diffusion assay, by exploiting the UV absorption at *λ* of 561 nm of the RhB covalently linked to the nano-PEG. In particular, a layered system was created consisting of an agarose gel, (which acts as an acceptor compartment), and on top a mucin dispersion layer in PBS (1 mg/mL). As depicted in Fig. 8C, the NiM_(Rapa/PEG)_ dispersion was placed on top of the mucin layer and subsequently, each vial was incubated at 37 °C; at defined time intervals, the mucin dispersion was removed, and the agarose gels were rinsed with distilled water, dissolved at 60 °C and analyzed by UV spectrophotometry. The figure also shows a photograph where it is possible to see the diffusion of the particles through the mucin layer in the first 50 min after incubation; only after 25 min, it is possible to observe how some particles have crossed the entire mucus layer and reached the agarose layer.

Transmittance values obtained from UV analysis, reported in Fig. 8D, showed that the amount of NiM_(Rapa/PEG)_ that diffuses through the mucus layer increases as a function of the incubation time and that, in the first 5-h incubation, this amount is significantly higher than NiM_(Rapa)_. Moreover, for either NiM_(Rapa/PEG)_ or NiM_(Rapa)_ samples, the more the incubation time increases, the more the transmittance is reduced, up to about one-third of the starting value after 24 h.

## Conclusions

In this work, we described the production of a powder formulation for the pulmonary administration of Rapamycin (Rapa), a powerful anti-inflammatory drug, whose local administration in the lungs could expand its use in therapy. In particular, the formulation was obtained by spray-drying and was made up of mannitol-based microparticles containing Rapa-loaded pegylated nanoparticles. The latter were obtained by the nanoprecipitation of the PHEA-g-RhB-g-SUCC-PCL-g-PEG graft copolymer, which was synthesized step by step from the α,β-poly(*N*-2-hydroxyethyl)-d,l-aspartamide (PHEA) by grafting of rhodamine B (RhB), a succinylated derivative of poly-ɛ-caprolactone (PCL-SUCC), and with polyethylene glycol (PEG). It has been shown that these microparticles possessed the aerodynamic properties suitable for administration by means of dry powder inhaler devices and that, once placed in contact with lung simulated fluids, were able to dissolve and release Rapa-loaded pegylated nanoparticles, potentially able to diffuse through the mucus. The mucus-diffusion capacity could be attributed to the presence of PEG on the surface, as showed by XPS analysis. Produced NiM were also able to release and protect Rapa in simulated lung and cell fluids.

## Abbreviations

Rapa: Rapamycin
PCL nano: Empty PCL-based nanoparticles
Nano: Empty PHEA-g-RhB-g-SUCC-PCL-based nanoparticles
Rapa-loaded nano: Rapa-loaded PHEA-g-RhB-g-SUCC-PCL-based nanoparticles
Nano-PEG: Empty PHEA-g-RhB-g-SUCC-PCL-g-PEG-based nanoparticles
Rapa-loaded nano-PEG: Rapa-loaded PHEA-g-RhB-g-SUCC-PCL-g-PEG-based nanoparticles
NiM: Nano- into micro-containing empty nano
NiM_(PEG)_: NiM containing empty nano-PEG
NiM_(Rapa)_: NiM containing Rapa-loaded nano
NiM_(Rapa/PEG)_: NiM containing Rapa-loaded nano-PEG
